# Icariin improves cognitive impairment by inhibiting ferroptosis of nerve cells

**DOI:** 10.18632/aging.205144

**Published:** 2023-10-24

**Authors:** Yang Yang, Yiming Fu, Zhipeng Qin, Hongyan Pei, Liping Zhai, Qiaobing Guan, Shasha Wu, Heping Shen

**Affiliations:** 1 Shenyang Medical College, Shenyang 110000, Liaoning Province, China; 2 Criminal Investigation Police University of China, Shenyang 110000, Liaoning Province, China; 3 College of Chinese Medicinal Materials, Jilin Agricultural University, Changchun 130118, Jilin, China; 4 The Second Affiliated Hospital of Jiaxing University, Jiaxing 314001, Zhejiang Province, China

**Keywords:** icariin, ferroptosis, Alzheimer's disease, MDM2, oxidative stress

## Abstract

Aim: We investigated the effect and mechanism of Icariin (ICA) on improving neurobehavioral ability of mice with Alzheimer's disease (AD).

Methods: We selected 10-month-old APP/PS1 mice (AD) and wild-type C57BL/6J mice (Normal). After intragastric administration of ICA, Morris water maze was employed to detect neurobehavioral improvements, and to assay key ferroptosis indicators and oxidative stress levels. The common target of ICA for resisting ferroptosis and AD was predicted by network pharmacology.

Results: ICA could improve the neurobehavioral, memory and motor abilities of AD mice. It could lower the ferroptosis level and enhance the resistance to oxidative stress. After inhibition of MDM2, ICA could no longer improve the cognitive ability of AD mice, nor could it further inhibit ferroptosis. Network pharmacological analysis revealed that MDM2 might be the target of ICA action.

Conclusions: We found that ICA can inhibit ferroptosis of nerve cells, thereby ameliorating neural damage in mice with AD.

## INTRODUCTION

Epimedium brevicornum Maxim has the effects of tonifying kidney yang, strengthening muscles and bones, as well as dispelling wind dampness [1]. Among a variety of its active constituents, Icariin (ICA) is a flavonoid compound extracted from Epimedium plants [2]. According to modern pharmacological studies, ICA has multifarious pharmacological effects such as anti-depression, improvement of ischemic brain injury, anti-dementia and anti-aging [3, 4]. Research on AD has found that ICA possesses certain effects, with diverse mechanisms. ICA can reduce Aβ deposition to improve the symptoms of AD model, and its mechanism of action may be associated with the regulation of PI3K/AKT [5]. In the nervous system, ICA can antagonize the damaging effect of neurotoxins on neurons in the rat cortex and hippocampus, and its mechanisms may be achieved by inhibiting the activation of microglia cells, reducing the production of inflammatory cytokines, alleviating the damage of inflammatory transmitters to neurons, and improving the learning and memory abilities of mice [6, 7]. According to current research findings, AD is a neurodegenerative disease related to multiple factors. ICA, as the major active constituent of traditional Chinese herbal medicine Epimedium brevicornum Maxim, has multifarious pharmacological activities and plays a significant role in multiple organs and tissues. However, the exact target of its action has never been reported, and relevant mechanism research appears superficial, which necessitates further investigation.

In the research of AD, it has been found that ferroptosis of nerve cells is a major event of nerve injury, and glial cell death also plays an important regulatory role in AD. Apart from conventional oxidative stress regulation, P53 is one of the important proteins that induce ferroptosis. P53 and MDM2 combine into a complex to regulate SLC11A7C and mediate lipid peroxidation [8, 9]. In this study, we attempted to further explain the role, exact mechanism and target of ICA in treating AD from the ferroptosis perspective.

## RESULTS

### ICA improves cognitive behavior and ferroptosis in AD mice

**Figure 1:**
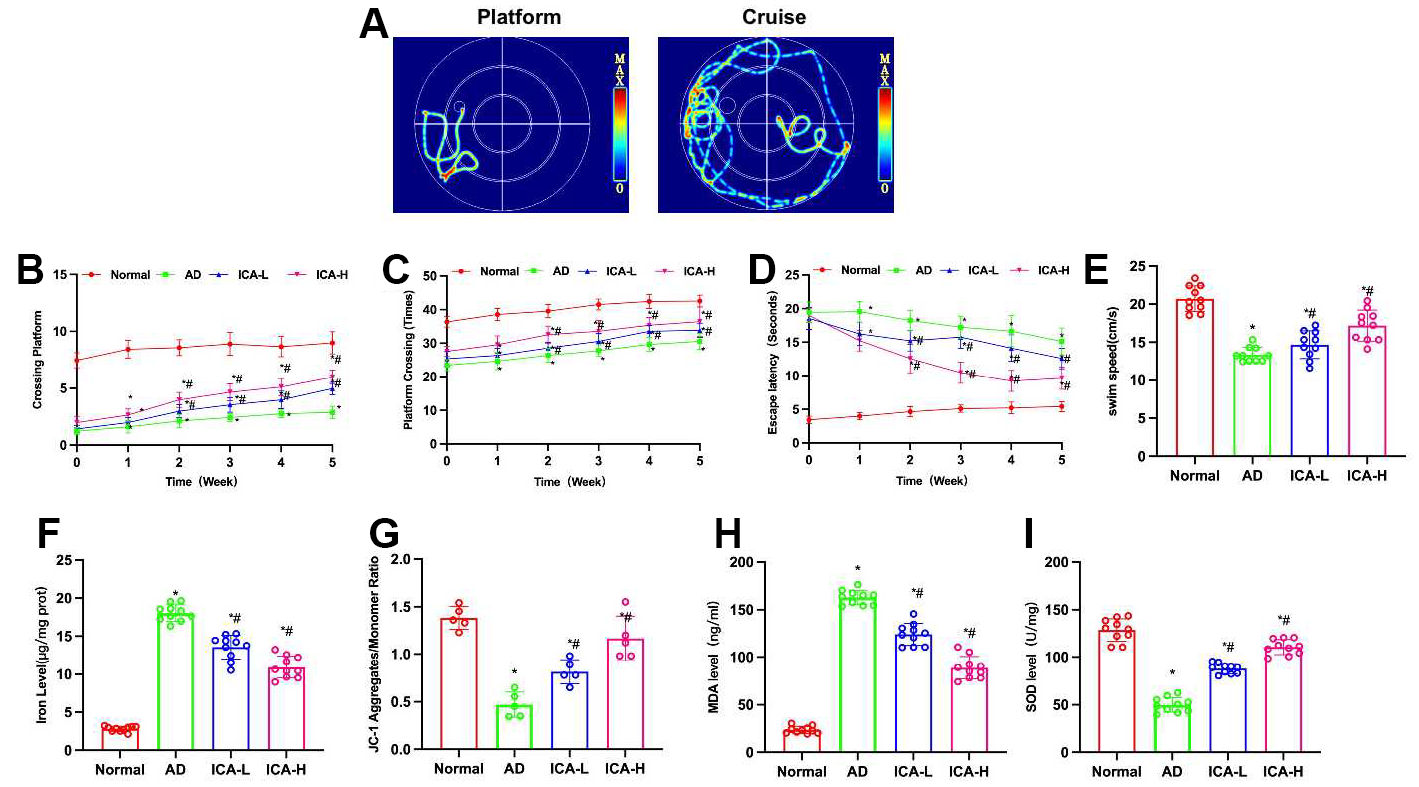
**ICA improves cognitive behavior in AD mice.** (**A**–**E**) Morris water maze (n= 10). Compared to the Normal mice, AD mice exhibited longer escape latency, fewer platform crossings and shorter swimming distance. ICA could shorten the escape latency, increase the number of platform crossings and prolong the swimming distance. ^*^P< 0.05 vs. Normal; ^#^P< 0.05 vs. AD. (**F**, **G**) Iron content and JC-1 (n=10). In AD mice, the polymer/monomer ratio was downregulated, while the iron ion content was upregulated. ICA could lower the iron ion content in the brain, enhance the polymerization level and increase the polymer/monomer ratio. ^*^P< 0.05 vs. Normal; ^#^P< 0.05 vs. AD. (**H**, **I**) SOD and MDA (n=10). Compared to the Normal mice, the AD mice exhibited downregulated SOD and upregulated MDA. ICA could resist oxidative damage, inhibit the MDA level and elevate the SOD level. ^*^P< 0.05 vs. Normal; ^#^P< 0.05 vs. AD.

According to the water maze results, ICA could improve the cognitive and behavioral abilities of AD mice. Compared to the Normal group, AD group exhibited obvious cognitive impairment, prolonged escape latency, reduced number of platform crossings and shortened swimming distance. ICA could improve such situation, which shortened the escape latency, increased the number of platform crossings and prolonged the swimming distance, showing significant differences from the AD group (Figure 1A–1E).

During the ferroptosis detection, it was found that the polymer/monomer ratio in JC-1 of AD mice was downregulated, while the content of iron ions was upregulated. ICA could lower the iron ion content in the brain, enhance the polymerization level and increase the polymer/monomer ratio, which differed significantly from the AD group (Figure 1F, 1G). As suggested by the oxidative stress indicators, compared to the Normal mice, AD mice exhibited SOD downregulation and MDA upregulation in the brain. ICA could resist oxidative damage, inhibit the MDA level and elevate the SOD level (Figure 1H, 1I).

### Network pharmacological analysis of ICA targets in AD and ferroptosis

**Figure 2:**
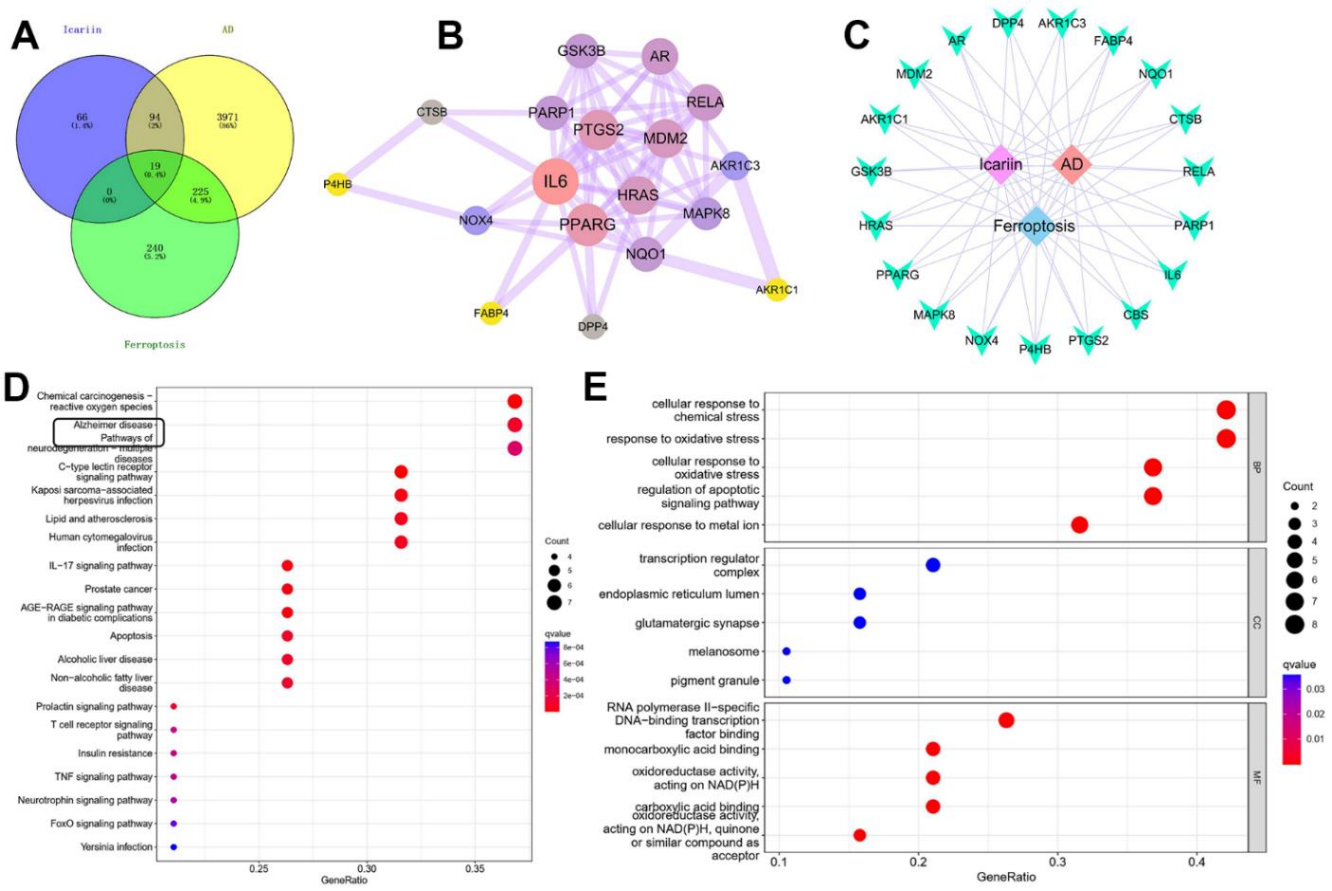
**Network pharmacological analysis of ICA targets.** (**A**, **B**) PPI network construction and topological analysis revealed that MDM2 may be a common target of ICA in AD and ferroptosis. (**C**) Construction and visualization analysis of “medicinal material–component–target” network. (**D**, **E**) GO and KEGG enrichment analyses.

There were 179 drug targets, 4,309 AD-related genes and 484 ferroptosis genes. The genes intersecting among the three totalled 19, accounting for 0.4% of the overall genes. Eighteen related genes were obtained from the PPI network of medicinal materials for disease treatment, and 64 targets were interrelated. We found that MDM2 was the primary target of ICA, which was closely associated with ferroptosis as well (Figure 2A, 2B). A “medicinal material–component–target” visual network diagram was created via Cytoscape (Figure 2C) as well as 107 signaling pathways like C-type lectin receptor, chemical carcinogenesis–reactive oxygen species and IL-17 pathways (Figure 2D, 2E).

### Inhibition of MDM2 can antagonize ICA’s effect on AD mice

**Figure 3:**
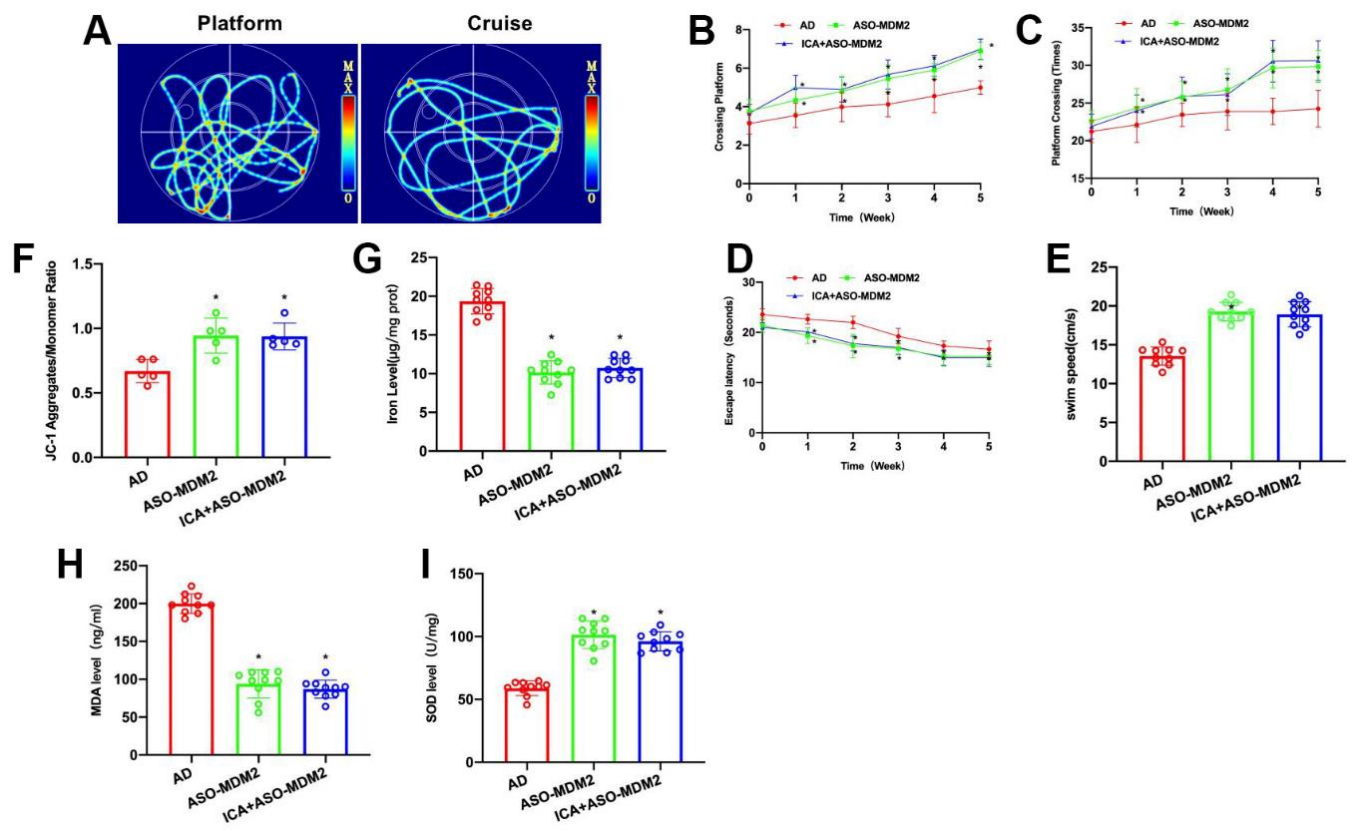
**Inhibition of MDM2 can antagonize ICA’s effect on AD mice.** (**A**–**E**) Morris water maze (n= 10). In ASO-MDM2 and ICA+ASO-MDM2 groups, escape latency was shortened, platform crossings were increased and swimming distance was lengthened, showing significant differences from the AD group, albeit insignificant differences between these two groups. ^*^P< 0.05 vs. AD. (**F**, **G**) Iron content and JC-1 (n= 10). ASO-MDM2 and ICA+ASO-MDM2 could lower the iron ion content in brain, enhance the level of JC-1 polymerization, and increase the polymer/monomer ratio. ^*^P< 0.05 vs. AD. (**H**, **I**) SOD and MDA (n= 10). ASO-MDM2 and ICA+ASO-MDM2 could suppress the level of MDA and elevate the level of SOD. ^*^P< 0.05 vs. AD.

To verify the correlation between ICA and MDM2, ASO-MDM2 was used in conjunction with ICA in animal experiments to inhibit the expression of MDM2 and to antagonize its effect. Morris water maze results demonstrated that ASO-MDM2 and ICA+ASO-MDM2 could improve the cognitive ability of AD mice, shorten their escape latency, increase their platform crossings and prolong their swimming distance, showing significant differences from the AD group, albeit insignificant differences between these two groups (Figure 3A–3E).

During the detection of ferroptosis index, it was found that ASO-MDM2 and ICA+ASO-MDM2 could lower the iron ion content in brain, elevate the level of JC-1 polymerization, and increase the polymer/monomer ratio (Figure 3F, 3G). Detection of oxidative stress index showed that ASO-MDM2 and ICA+ASO-MDM2 could suppress the level of MDA and elevate the level of SOD (Figure 3H, 3I).

### Validation of target relationship between ICA and MDM2

**Figure 4:**
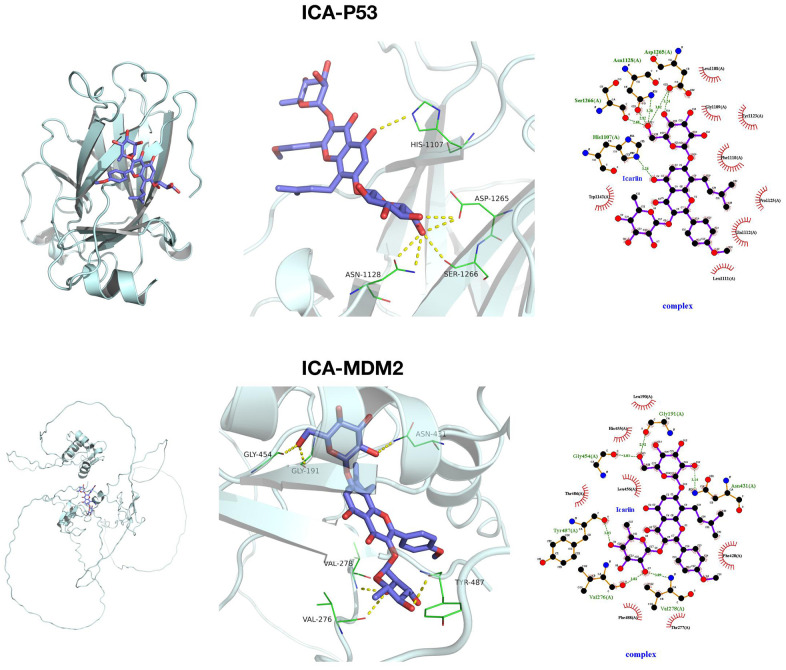
**Target relationship between ICA and MDM2.** Small molecule docking models of ICA, P53 and MDM2.

We docked ICA with P53 and MDM2 through small molecule–protein docking, finding that ICA and P53 were mainly assembled by hydrophobic bonds at HIS, ASP, ASN and SER sites, which were bound primarily with O and insufficiently tightly with hydrophobic pockets. Meanwhile, ICA and MDM2 were assembled by hydrogen and hydrophobic bonds at sites like GLY, VAL, TYR and ASN, which were bound tightly with hydrophobic pockets. From a docking structure perspective, ICA and MDM2 were more tightly bound, with lower binding energy (Figure 4).

## DISCUSSION

Modern pharmacological studies have shown that icariin (ICA), one of the effective constituents extracted from Epimedium brevicornum Maxim, has multifarious pharmacological properties such as cardiovascular protection, immune regulation, anti-tumor, anti-apoptosis and anti-inflammation [10, 11]. According to substantial recent studies, ICA has an obvious neuroprotective action, which can improve the learning and memory functions of AD model animals remarkably. The anti-AD effect of ICA has been found to be associated with the energy metabolism [12], repair of mitochondrial damage [13], neuronal apoptosis inhibition, and suppression of microglial cell activation [14]. In the event of abnormal energy metabolism, the following mechanisms may lead to the pathological changes of AD: massive production of reactive oxygen species, abnormal response of microglial cells, neuronal apoptosis and abnormal autophagy system. It has been found that ICA's improvement of cognitive function may be related to its protection of mitochondrial function and regulation of neuronal energy metabolism. Research has shown that in APP/PS1 double-transgenic mice, daily gavage administration of ICA compound could enhance the glutathione peroxidase (GSH-Px) and SOD activities [15], thereby improving the oxidative stress injury. ICA's ability to improve oxidative stress damage has also been confirmed through *in vitro* experiments. In the hydrogen peroxide-induced PC12 cell damage, ICA has been found to reduce the glutathione (GSH) consumption by weakening the secretion of lactate dehydrogenase (LDH), thereby preventing the DNA oxidative damage and inhibiting the subsequently activated caspase-3 and p53 [14, 16]. ICA can reduce the Aβ_25-35_-induced apoptosis of PC12 cells by inhibiting Tau hyperphosphorylation at Ser396, Ser404 and Thr205, which may be associated with its activation of PI3K/AKT pathway [17, 18]. To sum up, ICA can improve cognitive dysfunction caused by various factors, which is probably related to multiple mechanisms. Nonetheless, its specific interventive and protective mechanisms remain unclear, especially the exact target of action.

Ferroptosis, as a regulatory mode of cell death discovered in recent years, is driven by iron-dependent lipid peroxidation, which is the result of imbalance between cell metabolism and redox homeostasis [19, 20]. Features of ferroptosis, such as iron imbalance, accumulation of reactive oxygen species, decreased glutathione level and GPX4 inactivation, are also important pathological events of AD and cognitive dysfunction [21, 22]. Moreover, high-throughput sequencing has found that the ferroptosis-related differentially expressed genes were highly enriched in the AD-related gene set, indicating that ferroptosis is one of the new factors in the AD occurrence and development, which is also a new means of treating AD [23, 24]. In our current research on APP/PS1 mice, activation of ferroptosis was present in AD mice, and this model is applicable to the study of AD–ferroptosis. ICA could improve cognitive impairment and behavioral abnormalities in mice. Morris water maze revealed that ICA could ameliorate cognitive impairment in AD in a dose-dependent manner. ICA alleviates ferroptosis in AD primarily by inhibiting mitochondrial lipid peroxidation injury. We further applied network pharmacology to analyze the possible targets between AD, ferroptosis and ICA, finding that MDM2 was closely associated with ICA and was a crucial histone of P53 complex. It has been found that p53 can inhibit the SLC7A11 expression at the transcriptional level, thereby promoting cellular ferroptosis to allow tumor inhibition.

## CONCLUSIONS

Through network pharmacology combined with experimentation, we find that ICA can improve the cognitive and motor abilities of AD mice by inhibiting the ferroptosis of nerve cells. However, further exploration and research are needed on how ICA can be applied in clinical practice.

## MATERIALS AND METHODS

### Mouse interventions

Animal experimental protocols were reviewed and approved by the Ethics Committee of Jiaxing University. The animal experiments were in line with the relevant regulations of animal ethics and welfare. During the experiments, the mice lived, drank and ate in a unified environment without any behavior violating animal ethics and welfare. The mice were raised in the Laboratory Animal Center of Jiaxing University.

Ten wild-type C57BL/6J mice (Normal) and forty 10-months-old APP/PS1 double-transgenic AD mice (AD) were purchased from Model Animal Research Center of Nanjing University and raised in Laboratory Animal Center of Jiaxing University. Mice were divided into 4 groups (n = 10 per group): Normal, AD, ICA-L (5 mg/kg) and ICA-H (10 mg/kg). ICA was intragastrically administered to AD mice once daily. AD and Normal mice were given equal dose of normal saline for 5 consecutive weeks. During the mechanism research, we used 30 AD mice. The mice were divided into 3 groups (n = 10 per group): AD, ASO-MDM2 and ICA+ASO-MDM2. Mice in the ASO-MDM2 group were injected with 15 nmol of antisense MDM2 oligonucleotides (ASO-MDM2, Ribo Life Science, Beijing, China) once every 3 days through the tail vein for MDM2 inhibition. Mice in the ICA+ASO-MDM2 group were intragastrically administered with 10 mg/kg ICA once daily, in addition to the ASO-MDM2 injection as described in the ASO-MDM2 group. The intervention lasted for 5 consecutive weeks. After the 5th week, the mice underwent neurobehavioral tests and killed by CO_2_ asphyxia.

### Mouse behavioristics

To perform the Morris water maze test, the model EthoVision XT8.5 (Noldus) in our laboratory was used, which was equipped with EthoVision system camera, computer, as well as image acquisition and analysis software. The water in the maze was 2 cm above the platform and its temperature was controlled at 25° C. Experiment was conducted once a week. The training period was the first 5 days of the experiment, and the platform position was fixed. Every day, 4 rounds of tests were carried out for each mouse, which entered the water from different positions to train its spatial memory ability. The time for each test round was set as 90 s. If the mouse found the platform within the specified time and stayed on it for more than 3 s, the test would be terminated, and the system automatically recorded the time (latency) for mice to find the platform. If the mouse failed to find the platform within 90 s, it would be guided to the platform and let stay on it for 10 s. The latency for finding the platform was recorded as 90 s. On the 6th day, the platform was removed and only one round of test was conducted. All mice entered the water from the diagonal quadrant of the original platform, and the number of times they passed through the platform within 90 s was recorded, as well as the time they stayed in the quadrant where the platform was located and the distance they traveled.

### Iron level detection

We used the iron assay kit (Jiancheng Bioengineering Institute, Nanjing, China) to detect the iron levels in mouse brain tissues and cells. After grinding with liquid nitrogen, the mouse brain tissues were treated with NP-40. During the cellular experiment, PC12 was washed with PBS and then also treated with NP-40 on ice for 30 min. Following centrifugation of tissue homogenate and cell fluid at 2,500 g, the supernatants were collected, added sequentially with double distilled water, 2 mg/L standard iron solution and color developing agent as per the instructions, boiled in water bath for 5 min, cooled and centrifuged at 3,500 g, followed by measurement of the OD value at 520 nm. Iron level (μmol/gprot) = (A sample - A blank/A standard - A blank x standard concentration)/protein concentration (gprot/L).

### SOD and MDA assays

Total superoxide dismutase (SOD) and lipid oxidation (MDA) were detected via SOD assay kit (Jiancheng Bioengineering Institute, Nanjing, China) and MDA assay kit (Beyotime Biotechnology, Shanghai, China). Tissues and cells were treated as described in previous section. Protein levels in tissues and cells were quantified by BCA assay, and the experiment was conducted after adjusting the protein levels. The supernatant was added with reagent as per the kit instructions and incubated at 37° C for 20 min, followed by measurement of OD value at 450 nm. Finally, SOD was expressed as U/mgprot, while MDA was assayed the same as SOD and expressed as μmol/mg.

### Statistical processing

All measurement data were expressed as $\left(\bar{x}\pm \text{s}\right)$, and analyzed and processed via SPSS 17.0. After homogeneity of variance test, analysis between two groups of data was made by two independent samples t-test. One-way ANOVA was employed to analyze three or more groups of data, and LSD method was employed for subsequent pairwise comparison between groups. All of the above tests were two-sided, and differences were considered statistically significant when P< 0.05.

### Availability of data and materials

The data that support the findings of this study are available from the corresponding author upon reasonable request.

### Consent for publication

All authors approve the publication of the article.
